# Supplementary figures and images for: Quantitative Computed Tomography Lung COVID Scores with Laboratory Markers: Utilization to Predict Rapid Progression and Monitor Longitudinal Changes in Patients with Coronavirus 2019 (COVID-19) Pneumonia
Source: Biomedicines. 2024 Jan 6;12(1):120. doi: 10.3390/biomedicines12010120 (PMC10813449; doi:10.3390/biomedicines12010120)

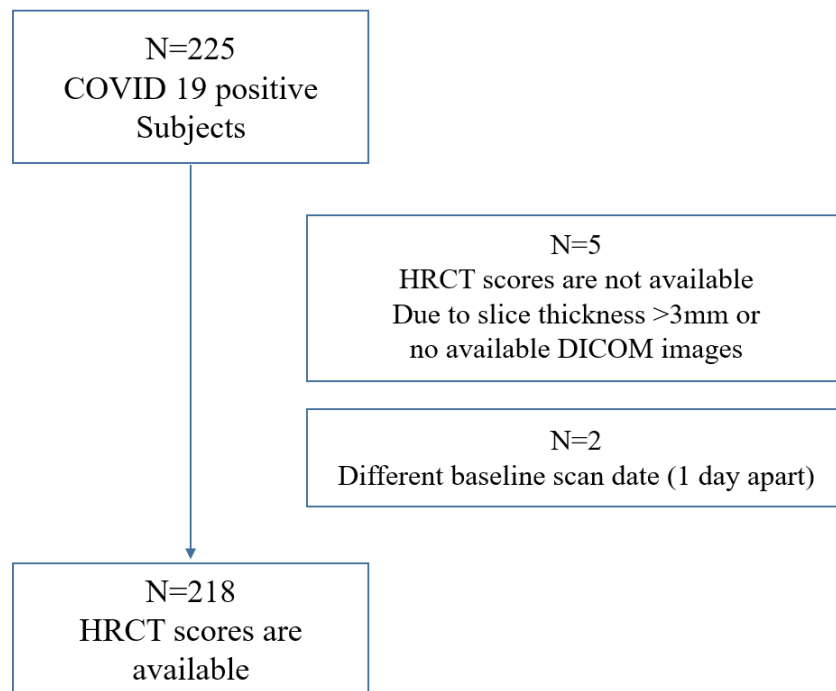

**Figure S1.** Study population.

Supplement: Supplementary file 1 [file biomedicines-12-00120-s001.zip › biomedicines-2778174-supplementary.pdf]
